# Social Imitation of Alcohol Consumption and Ingratiation Motives in Young Adults

**DOI:** 10.1037/adb0000150

**Published:** 2016-06

**Authors:** Eric Robinson, Melissa Oldham, Maxine Sharps, Alexandra Cunliffe, Jade Scott, Emma Clark, Katie Piercy, Matt Field

**Affiliations:** 1 Psychological Sciences, Institute of Psychology, Health & Society, University of Liverpool and UK Centre for Tobacco and Alcohol Studies; 2 Psychological Sciences, Institute of Psychology, Health & Society, University of Liverpool; 3 Psychological Sciences, Institute of Psychology, Health & Society, University of Liverpool and UK Centre for Tobacco and Alcohol Studies

**Keywords:** alcohol, social drinking, mimicry, ingratiation, need to belong

## Abstract

Across 2 studies we tested the hypothesis that social ingratiation motives may be an important factor explaining social imitation of alcohol consumption. In Study 1, participants drank alcohol with a heavy versus light drinking confederate under conditions that were designed to heighten or reduce (participants believed they would not be judged) motivation for ingratiation. In Study 2 we manipulated the degree to which participants believed they had already successfully ingratiated themselves with a heavy or no (alcohol) drinking confederate. In Study 1, participants’ alcohol consumption was most strongly influenced by the confederate’s drinking behavior when they believed that they would later be judged by the confederate. In Study 2, participants’ alcohol consumption was influenced by the confederate’s drinking behavior and this effect was particularly pronounced if participants were unsure if the confederate had accepted them. The desire for social ingratiation may in part explain why people imitate the drinking behavior of those around them.

Social context exerts a strong influence on alcohol consumption (Oostveen, Knibbe, & de Vries, 1996; Quigley & Collins, 1999; Robinson, Jones, Christiansen, & Field, 2015) and as the number of peers present during drinking increases, so does the amount of alcohol each person consumes (Thrul & Kuntsche, 2015). There is also convincing evidence for social imitation of alcohol consumption: drinking with heavy drinking partners increases alcohol consumption (Dallas et al., 2014; Larsen, Engels, Granic, & Overbeek, 2009; Larsen, Engels, Souren, Granic, & Overbeek, 2010). The mechanisms behind social imitation of alcohol consumption are ambiguous (Dallas et al., 2014; Larsen, Engels, Granic, & Huizink, 2013). However, recent findings suggest that social bonding may in part explain why mimicry of alcohol consumption occurs. For example, a genetic predisposition that is associated with social adaptation of alcohol consumption (Larsen et al., 2010; Mrug & Windle, 2014) has also been found to increase the likelihood that a person experiences social bonding when drinking with others (Creswell et al., 2012).

Because social mimicry is thought to be a strategy that can facilitate bonding and interpersonal closeness (Lakin & Chartrand, 2003), people may mimic the actions of others to ingratiate themselves (Chartrand & Bargh, 1999; Chartrand & Lakin, 2013). Although it has been theorized that social ingratiation motives may explain social mimicry of alcohol consumption (Dallas et al., 2014), there has been no direct examination of this proposition. In research examining food consumption, there is some indication that the desire to be liked by a dining partner influences social mimicry of food intake, whereby the quality of social interaction (Hermans, Engels, Larsen, & Herman, 2009) and feelings of social acceptance (Robinson, Tobias, Shaw, Freeman, & Higgs, 2011), predict the degree to which a person copies the eating behavior of a present dining partner. Likewise, the extent to which a fellow diner’s food intake is perceived as conveying a socially “appropriate” amount to eat is predictive of social influence on eating (Vartanian, Sokol, Herman, & Polivy, 2013). In the alcohol literature, it has also been shown that individuals with a high need for social acceptance are more likely to be influenced by the drinking behavior of a peer (Caudill & Kong, 2001). Thus, we reason that rather than being a passive conformity process, the tendency for a person to socially mimic alcohol consumption may actually be dictated by social ingratiation motives. According to this social ingratiation account, mimicry of alcohol consumption is predicted to be most likely to occur when social ingratiation motives are heightened (Lakin, Chartrand, & Arkin, 2008) and may be less likely to occur when there is little reason to ingratiate oneself with a fellow drinker (Lakin, Jefferis, Cheng, & Chartrand, 2003). These predictions are based on the premise that behavioral mimicry is used to achieve affiliation goals and once affiliation has been achieved, mimicry is no longer adaptive. For example, behavioral mimicry of hand gestures has been shown to be most pronounced when a person is primed with a need for social affiliation, and mimicry is reduced once that need has been met (Lakin & Chartrand, 2003).

The aim of the present studies was to test a social ingratiation account of social mimicry of alcohol consumption. In Study 1 participants drank with a heavy (or light) drinking confederate under conditions that were designed to heighten or remove their motivation to ingratiate with that person (participants were led to believe they would or would not later be judged by the confederate). In Study 2 we manipulated the degree to which participants believed they had already successfully ingratiated themselves with a heavy (or no) drinking confederate. We hypothesized that participants would mimic the alcohol consumption of a confederate after experimental manipulations that were designed to heighten their need for ingratiation. We predicted that social mimicry of alcohol consumption would be reduced under experimental manipulations that were designed to reduce participants’ need for ingratiation.

## Study 1

### Method

#### Overview

Participants took part in a study about “meeting new people” that involved drinking with a peer (confederate) who consumed either a large or a small amount of alcohol. Because evaluation by others increases a person’s desire to make a good impression and be liked (Baumeister & Leary, 1995; Leary, Tchividijian, & Kraxberger, 1994), participants were led to believe either that the confederate would later evaluate them, or that they would be evaluating the confederate. Given that people mimic the behavior of others to foster liking and achieve ingratiation (Lakin & Chartrand, 2003; van Baaren, Janssen, Chartrand, & Dijksterhuis, 2009), we predicted that participants would mimic the confederate when they believed they would later be evaluated by them (heightened ingratiation motivation), but they would be less likely to mimic the confederate when they believed they would later be evaluating the confederate (reduced ingratiation motivation).

#### Participants

Eighty (58 women) first-year undergraduate psychology students (*M* age = 19.2 years, *SD* = 1.9) participated for course credit (participants could choose from a number of studies, including this one, to obtain their credits). Potential participants were screened over email and had to be aged 18 years or older, fluent in English, regular drinkers of alcohol (weekly), not pregnant, not using medication likely to affect drinking alcohol, and have no history of alcohol-use disorder. Studies 1 and 2 were approved by the University of Liverpool research ethics committee.

#### Design

Participants were randomized into a 2 × 2 between-subjects design. Independent variables were ingratiation motive (heightened or reduced) and confederate drinking level (heavy or light drinking). The confederate was a 20-year-old female undergraduate psychology student unknown to participants (she was in a different year group) who behaved as a normal participant throughout the study. In both studies we used only female confederates on the basis of previous work that indicates that confederate-participant sex differences do not appear to moderate social influence on alcohol consumption (Larsen et al., 2012).

#### Questionnaire measures

Participants completed a number of questionnaires after the experimental manipulation. First, participants provided demographic information before they were given information about the number of units of alcohol in common drinks and asked to estimate how many units of alcohol they consumed in a typical week. To distract from the true aims of the study, participants then completed seven filler items measuring their current mood (10 cm visual analogue scales), 10 mock personality questions (e.g., “I like to gossip at times”) and 19 questions that involved rating the confederate on a variety of adjectives (agree, disagree or unsure response format, e.g., “Hardworking”). Next, participants completed seven questions concerning their experience in the study (5-point Likert scale; 1 = *strongly disagree* to 5 = *strongly agree*). Some of these acted as filler questions to disguise our main variables of interest (e.g., “I found the task difficult”), but we also included questions to probe participant suspicion (see Supplemental Materials). After this, participants were asked to write down what they thought the aims of the study were (free text). Finally, we included questions to measure whether participants had noticed the amount of alcohol the confederate had consumed (see Supplemental Materials).

#### Procedure

Participants were instructed to attend a designated waiting area, where the confederate was waiting. When the participant arrived, the researcher escorted both the confederate and the participant to a laboratory that consisted of a table with two chairs facing each other. After participants had provided written informed consent, the researcher breathalyzed both participants (all participants’ blood alcohol content was 0%) and then explained the study procedures. The researcher informed participants that the study involved making judgments about another person. In the heightened ingratiation motive condition, the researcher informed participants that the confederate would be asking the real participant questions, and later completing questions about their first impressions of them. In the reduced ingratiation motive condition, the researcher stated that the real participant would be the one asking the confederate questions and reporting their first impressions. The researcher then explained that the person asking questions would be provided with a list of questions and that during this, both participants would be provided with an alcoholic drink (vodka and diet coke), to match the environment in which people often meet for the first time. The researcher then explicitly informed both participants that they were free to drink as much or as little of the drink as they desired. The real participant was provided with 125 ml of vodka and diet coke (25 ml of 37.5% vodka) and the confederate appeared to also receive the same drink (although their drink contained only diet coke). The confederate or real participant was then provided with the list of questions (e.g., “Where did you grow up?”) and the researcher left the room for 5 min. The confederate either drank all of their drink at a steady rate across the 5 min (heavy drinking) or they took a small sip at the start and subsequently did not drink any more of the drink (light drinking). In advance of experimental sessions, the confederate learned a script so that they would answer their questions in a consistent manner across all participants. Moreover, they were trained to not draw attention to the amount that they or the participant was drinking (e.g., they did not talk about or gesture to the participant’s drink or their own), and we ran several pilot sessions with the confederate to ensure that their behavior toward participants was neutral and consistent. When the researcher returned, the confederate and the participant were separated into different rooms, to allow the participant to complete the *Questionnaire Measures* and to be breathalyzed. The participant was then debriefed and thanked.

#### Data analysis

Our main analysis of interest was a 2 (ingratiation condition) × 2 (confederate drinking) analysis of variance (ANOVA; dependent variable: milliliter of drink consumed). We used the same strategy to examine whether participants allocated to different experimental conditions were well matched on their typical alcohol consumption and age, and we used χ^2^ to determine if conditions were balanced for gender.

### Results

#### Participants

Experimental groups were well matched on age and typical alcohol consumption (all *p*s > .05). Gender was similarly distributed across the confederate drinking conditions, but there was a higher proportion of males in the heightened ingratiation motive condition (15 men, 26 women) than in the reduced ingratiation condition (5 men, 32 women; *p* < .05). We added gender as an additional factor in our main analysis of interest. Gender was not directly associated with alcohol consumption, it did not interact with any of the independent variables (*p*s > 0.20) and it did not change the pattern of any results when included as a covariate, so our reported results do not include gender as a factor. See Table 1 for age, gender, and typical alcohol consumption across conditions.[Table-anchor tbl1]

#### Consumption

There was a significant main effect of confederate drinking behavior, *F*(1, 76) = 17.1, *p* < .001, η_p_^2^ = .18, in which drinking with a heavy drinking confederate increased alcohol consumption. There was no significant main effect of ingratiation motive, *F*(1, 76) = 0.01, *p* = .99, η_p_^2^ = .01, but there was a significant interaction between confederate drinking behavior and ingratiation motives, *F*(1, 76) = 6.3, *p* = .014, η_p_^2^ = .08. Because our interest was in the strength of the mimicry effect (heavy drinking vs. light drinking confederate), to follow up this interaction, we examined the effect of confederate drinking behavior under heightened versus reduced ingratiation motives, using separate planned pairwise comparisons (*t* tests). When participants drank under heightened ingratiation motives they drank more in the presence of a heavy drinking confederate versus a light drinking confederate, *t*(40) = 4.9, *p* < .001, *d* = 1.5, and this effect remained significant after Bonferroni correction (*p* < .001). When participants drank under reduced ingratiation motives, there was no significant effect of confederate behavior, *t*(36) = 1.1, *p* = .28, *d* = 0.36, Bonferroni corrected *p* = .56. See Table 2 for Condition means and standard deviations. As expected, under conditions of heightened ingratiation, participant consumption was more similar to that of the confederate, than under conditions of reduced ingratiation.[Table-anchor tbl2]

#### Demand characteristics

As expected, the confederate was not known to participants (see Supplemental Materials). No participants correctly guessed the aims of the study. Eleven participants came close to guessing the aims; that is, reporting some suspicion about whether the study concerned how much alcohol they consumed or the influence of the confederate (e.g., “to see how the presence of someone else influenced how much I drank”). Removal of these participants did not alter the results reported above (see Supplemental Materials).

### Discussion

In Study 1, participants who were led to believe that they would subsequently be judged by a confederate (heightened ingratiation concerns) were more likely to imitate the drinking behavior of that confederate. A limitation of Study 1 was the low number of male participants and gender imbalance across conditions. Moreover, our experimental manipulation meant that cognitive load may have been greater for participants in the reduced ingratiation motives condition (because they believed that they had to form an impression of the confederate and report this back to the experimenter), and in principle this could have interfered with their capacity to mimic the confederate’s drinking behavior. In addition, Study 1 had a relatively small sample size. Thus, the lack of statistically significant mimicry effect under conditions of reduced ingratiation (*p* = .28, *d* = 0.36) could be attributable to a lack of statistical power. Finally, the design of Study 1 meant that the confederate could not be blinded to the ingratiation condition that the participant had been assigned to, and it is feasible that this could have affected how they interacted with the participant. We addressed these issues in Study 2; we recruited a larger sample and did not limit our recruitment to psychology undergraduate students, which resulted in a larger number of male participants. In addition, we ensured that participants in different ingratiation conditions completed the same experimental task and confederates were blinded to the ingratiation condition each participant was assigned to.

## Study 2

According to a social ingratiation motive account of mimicry (Lakin et al., 2008, 2003), social influence on alcohol consumption should be observed when a person has yet to be socially accepted by a drinking partner (heightened ingratiation motives), but this mimicry should be reduced if a person believes that they have already successfully ingratiated themselves. In Study 2, we tested this prediction and adopted a social acceptance paradigm used previously by (Lakin & Chartrand, 2003; Lakin et al., 2008), in which we made participants feel socially accepted by a confederate (or not) before drinking together. To increase our confidence in the ecological validity of our findings regarding ingratiation motives, testing took place in either a naturalistic drinking environment (a “bar lab”) or the same standard laboratory as in Study 1. Based on the findings of Study 1, we predicted that when participants had not yet been socially accepted by the confederate they would mimic their alcohol consumption, but when they had already been accepted by the confederate, social mimicry may be less pronounced.

### Method

#### Overview

Participants took part in a study about social problem solving, while drinking with a confederate who consumed either a large amount of alcohol or none at all (i.e., they chose and consumed a soft drink beverage).^1^ Through the use of a bogus questionnaire, and before drinking together, participants were led to believe either that the confederate liked them and enjoyed their company (reduced ingratiation motivation condition), or that the confederate was unsure how much they liked them (heightened ingratiation motivation condition).

#### Participants

There were 149 (92 women) participants recruited from staff and students at the University of Liverpool (*M* age = 26.4 years, *SD* = 10.7). Participants were screened to ensure they met inclusion criteria as in Study 1. We powered the study (80% power) using GPOWER 3.1 to detect a significant medium sized interaction effect (as in Study 1) and recruited slightly above the required sample (*N* = 128), to account for any participants guessing the aims of the study.

#### Design

Participants were randomized into a 2 × 2 between-subjects design, with factors of Ingratiation motive (heightened or reduced) and confederate drinking level (heavy drinking or no drinking). Because of constraints on laboratory space, the first 80 participants completed the study in a mock bar laboratory (see Dallas et al., 2014, for more information about this laboratory). The remaining 69 participants completed the study in a laboratory similar to Study 1. Different researchers and confederates were used (all female, aged 20–21) across the two settings, although the exact same procedure was used in both settings.

#### Measures

##### First Impressions Questionnaire

Participants provided demographic information and made a series of ratings about their first impressions of the study (5-point Likert scale) and the confederate: “Based on first impressions . . . do you think the other participant is the type of person you could be friends with/will you enjoy spending time with the other person/do you think the other person is interesting?” *First Impressions Questionnaire (bogus):* to manipulate whether participants felt as though the confederate did or did not accept them, participants were exposed to a version of the *First Impressions Questionnaire* that appeared to have been filled out by the confederate. In the reduced ingratiation motives condition the questionnaire responses indicated that the confederate accepted the participant (e.g., strongly agree or agree to the three interpersonal items). In the heightened ingratiation motives condition the questionnaire responses indicated that the confederate had not accepted the participant (e.g., unsure or disagree). *Postdrinking Questionnaire:* As in Study 1, participants completed measures of their typical alcohol consumption, before answering questions about their experience in the study (see Supplemental Materials) and wrote down what they thought the aims of the study were. *Manipulation Check Questionnaire:* As a manipulation check, participants were asked to recall what drinks the confederate had chosen, as well as being asked to reproduce the bogus questionnaire responses they had earlier been exposed to (see Supplemental Materials).

#### Procedure

On arrival, the researcher escorted both the confederate and the participant to the laboratory. After providing informed consent the researcher left the confederate and real participant alone for 2 min. During this time the confederate initiated conversation with the participant (e.g., “Do you work at the university?”). On their return, the researcher explained to the participants that they were required to be breathalyzed and to complete a short questionnaire alone (*First Impressions Questionnaire*). The researcher then took the confederate to another laboratory. After a short delay, the researcher returned to the main laboratory and commented that they had left the breathalyzer. When retrieving the breathalyzer the researcher placed a stack of questionnaires on the table in view of the real participant, with the *Bogus First Impressions Questionnaire* on top of the pile and in view, before leaving to breathalyze the confederate. The confederate was blinded to whether the bogus questionnaire indicated that the confederate had responded positively about the participant (reduced ingratiation motive) or with uncertainty (heightened ingratiation motive). The researcher then returned shortly afterward to breathalyze the real participant and then asked them to complete their own version of the *First Impressions Questionnaire*. At this point the researcher “noticed” that they had left the confederate’s questionnaire on the table by mistake and removed it.

Next, the researcher returned the confederate to the main laboratory. The researcher then explained to the real participant and confederate that the main task would involve problem-solving. As in a previous study (Dallas et al., 2014), participants were provided with a questionnaire pack consisting of sets of four images and an anagram related to the images. The researcher explained to the participants that while completing the task they would be offered alcoholic and nonalcoholic drinks from a menu. The alcoholic drinks were a 275 ml bottle of lager (1.6 units/12.8 g of alcohol), a 125 ml glass of white wine (1.5 units/12 g of alcohol), and 25 ml of vodka with 100 ml of orange juice, diet coke, or diet lemonade (0.9 units/7.2 g of alcohol). The nonalcoholic options were water, orange juice, diet coke, or diet lemonade. The researcher first asked the confederate if they would like a drink and then the real participant. After approximately 10 min, the researcher returned and offered participants another drink. In the heavy drinking condition, the confederate chose vodka and diet coke for both drinks, but was actually served diet coke. In the no drinking condition, the confederate ordered diet coke for both drinks. Participants were able to drink for approximately 35 min. After completing the task, the researcher again separated the participants, and the real participant completed the *Postdrinking Questionnaire* and the *Manipulation Check Questionnaire*. Finally, the real participant was breathalyzed and debriefed.

#### Data analysis

We used a similar analysis strategy as in Study 1, although the primary dependent variable was grams of alcohol consumed. Because participants completed the study in a mock bar laboratory or a normal laboratory, we used a 2 × 2 × 2^2^ between-subjects design in our main planned analyses.

### Results

#### Participants

Conditions did not differ according to typical alcohol consumption or gender (all *p*s >.05). There was a tendency for participants tested in the bar laboratory to be younger than participants in the normal laboratory (see Table 3). However, age was not associated with our dependent variable and controlling for it in analyses had no effect on the results reported (see Supplemental Materials), so age was not included in the main analyses reported below.[Table-anchor tbl3]

#### Consumption

There was a significant main effect of confederate drinking behavior (*F*[1, 141] = 18.2, *p* < .001, η_p_^2^ = .11), whereby drinking with a heavy drinking confederate increased alcohol consumption. There was no significant main effect of ingratiation motives (*F*[1, 141] = 0.7, *p* = .42, η_p_^2^ = .005), although, as hypothesized, there was a significant interaction between confederate drinking behavior and ingratiation motives (*F*[1, 141] = 4.0, *p* = .048, η_p_^2^ = .027). Note: The three-way interaction between ingratiation motives, confederate drinking behavior, and laboratory type was not significant (see Supplemental Materials). Because our interest was in the strength of any mimicry effects, to follow up the confederate drinking behavior and ingratiation motive interaction, *t* tests were used. In the heightened ingratiation motive condition, participants drank more with a heavy versus no drinking confederate (*t*[72] = 4.3, *p* < .001, *d* = 1.0), and this effect remained significant after a Bonferroni correction (*p* < .001). In the reduced ingratiation motive condition there was a significant, but statistically smaller effect of confederate behavior (*t*[73] = 2.1, *p* = .045, *d* = 0.47), although the statistical significance of this effect was removed with Bonferroni correction (*p* = .09; see Table 4).[Table-anchor tbl4]

#### Demand characteristics

As expected, the confederate was not known to participants. No participants directly guessed the aims of the study. Twenty-four participants reported some suspicion about whether the study concerned the drink choice of the confederate or the bogus questionnaire (e.g., “to study social drinking”). Removal of these participants had little influence on the effect size of the confederate Drinking Behavior × Ingratiation Motive interaction, but it did result in the interaction only approaching statistical significance (*p* = .089, η_p_^2^ = .025). No other significant or nonsignificant effects in any analyses were affected by the removal of these participants (see Supplemental Materials).

## General Discussion

Across two studies we found that participants mimicked a confederate’s alcohol consumption when they were motivated to ingratiate themselves with the confederate. In Study 1, participants drank more when exposed to a heavy drinking confederate when they believed that person would later judge them, but they did not significantly adapt their alcohol consumption to match that of the confederate when they believed they would not be judged. In Study 2, participants imitated the alcohol consumption of a confederate if it was unclear whether they had ingratiated themselves with the confederate. The size of this mimicry effect was reduced when participants had been led to believe they had already ingratiated themselves with the confederate. Thus, rather than being “passively” socially influenced while drinking, the present studies suggest that social ingratiation motives may be a key reason why people copy the alcohol consumption of fellow drinkers.

The present findings are in line with research that suggests that one function of imitation is to increase social ingratiation and bonding (Chartrand & Bargh, 1999; Chartrand & Lakin, 2013; Robinson et al., 2011). The studies presented here provide the first empirical evidence that social ingratiation motives can contribute to social mimicry of alcohol consumption. Of particular note was our finding that participants were most likely to adapt their drinking behavior to that of a confederate when they believed that person was unsure of how much they liked them. This finding is in line with a number of findings from behavioral mimicry (e.g., Lakin et al., 2008; Over & Carpenter, 2009) and underlines the key role that ingratiation motives are likely to play in social mimicry of alcohol consumption.

Our findings also suggest that contexts or settings that result in heightened ingratiation concerns may render a person more susceptible to peer drinking influence (Litt, Stock, & Lewis, 2012). For example, peer influence on alcohol use in adolescents is well-recognized (Burk, Van der Vorst, Kerr, & Stattin, 2012; Ennett et al., 2006) and adolescents are thought to be particularly susceptible to peer influence on “risky” behaviors (Gardner & Steinberg, 2005; Steinberg & Monahan, 2007). Because adolescence is a life-period when social ingratiation concerns are heightened (Steinberg & Monahan, 2007), it may be the case that social ingratiation motives can explain age related differences in social influence on risky behaviors, such as alcohol use. Likewise, our findings corroborate those from a previous study that showed that individual differences in the need to belong were predictive of social mimicry of drinking behavior (Caudill & Kong, 2001). Thus, it may be the case that personality traits associated with heightened social ingratiation concerns explain why some people will be more susceptible to social mimicry of alcohol consumption than others.

Across both studies social mimicry effects under experimental conditions designed to minimize participant ingratiation motives were reduced. This finding is entirely consistent with our predictions and findings from studies that examined other types of behavioral mimicry, for example (Lakin & Chartrand, 2003). How these findings correspond to and explain previous examinations of social mimicry of drinking warrants some consideration. The influence that a drinking partner’s behavior has on one’s own alcohol consumption in an experimental setting is well replicated (Caudill & Kong, 2001; Dallas et al., 2014; Larsen et al., 2009; Quigley & Collins, 1999). Based on the present studies, we speculate that when social mimicry of drinking does occur, it is likely to be strongly driven by ingratiation motivation. Experimental studies of social drinking have tended to pair participants with an unknown confederate and although efforts have been made to study naturalistic drinking (e.g., the use of “bar” laboratories), it is likely that drinking with a stranger in a laboratory results in a scenario in which ingratiation and self-presentation concerns will be prominent.

As noted, in Study 2 we hypothesized that if participants were unsure if they had been accepted by a confederate, social mimicry of their alcohol consumption would occur, and our findings supported this hypothesis. A competing hypothesis is that if a person feels rejected this may trigger antisocial or aggressive behavior (Maner, DeWall, Baumeister, & Schaller, 2007; Twenge, Baumeister, DeWall, Ciarocco, & Bartels, 2007) or even an overall increase in alcohol consumption, rather than mimicry. We did not observe this. However, it is important to note that in Study 2 participants were not overtly rejected, instead they were led to believe that the confederate was unsure of how well they would get along. Thus, it seems likely that the level of social “rejection” and likelihood of being able to gain acceptance in future are important factors which determine when the presence or absence of social acceptance results in mimicry of drinking behavior. Further work specifically examining this would now be informative.

Alcohol consumption was examined in a controlled laboratory environment and with participants drinking with an unknown peer (a confederate). Although we know that social adaptation of alcohol can occur in naturally occurring drinking dyads (Dallas et al., 2014) and in naturalistic settings outside of the laboratory (Bot, Engels, Knibbe, & Meeus, 2007; Larsen, Overbeek, Granic, & Engels, 2012), the extent to which ingratiation motives explain social adaptation of drinking among friends in the “real world” now warrants attention. Moreover, future work could also examine whether ingratiation concerns specifically promote imitation of heavy drinking and/or light drinking peers, by including an additional experimental condition in which a confederate or peer drinks an “intermediate” amount of alcohol. In Study 1 we found that under experimental conditions designed to reduce ingratiation motives, social mimicry of alcohol consumption was reduced (in comparison with heightened ingratiation) and there was no statistically significant evidence of mimicry. It is possible that with a larger sample size there would have been a statistically significant effect of social mimicry under the reduced ingratiation motives condition, as we found in Study 2. This seems plausible because our manipulations may not have completely removed concerns about ingratiation in all participants. Indeed, although we based our ingratiation motives experimental conditions on previous research, we did not include formal measures of state ingratiation motives in the two studies. Inclusion of such measures during a study may influence participant behavior, but testing of the specific hypothesis that activation of ingratiation motives predicts mimicry of drinking behavior is an important issue for future research. Finally, we note that the researchers who administered questionnaires and provided drinks were not blinded to experimental condition in either study. While the confederates were trained to maintain consistency in behavior across participants, it was not possible to blind the confederates to participants’ ingratiation condition in Study 1, although confederates were blinded in Study 2. The consistent findings across Studies 1 and 2 suggest that confederate blinding is unlikely to account for our findings, and our findings on participant awareness suggest that demand characteristics did not play an important role in either study. Nonetheless, it is important to replicate these findings while ensuring that both researchers and confederates are fully blinded to participants’ allocation to experimental groups.

## Conclusions

The desire for social ingratiation may in part explain why people imitate the drinking behavior of those around them.

## Supplementary Material

10.1037/adb0000150.supp

## Figures and Tables

**Table 1 tbl1:** Study 1: Participant Characteristics by Condition

	Heightened ingratiation condition (*N* = 42)		Reduced ingratiation condition (*N* = 38)	
	Heavy drinking confederate (*N* = 21)	Light drinking confederate (*N* = 21)	Heavy drinking confederate (*N* = 19)	Light drinking confederate (*N* = 19)
Age (in years)	18.9 (.9)	19.4 (1.9)	19.0 (.8)	19.3 (3.2)
Units per week	11.5 (6.9)	13.5 (1.6)	12.0 (.5)	10.4 (9.3)
Gender	13 women, 8 men	13 women, 7 men, 1 n/a	15 women, 3 men, 1 n/a	17 women, 2 men
*Note.* Parentheses denote standard deviation for age and units per week.				

**Table 2 tbl2:** Study 1: Alcohol Consumption by Condition

	Heightened ingratiation condition (*N* = 42)		Reduced ingratiation condition (*N* = 38)	
	Heavy drinking confederate (*N* = 21)	Light drinking confederate (*N* = 21)	Heavy drinking confederate (*N* = 19)	Light drinking confederate (*N* = 19)
Mean milliliters consumed (*SD*)	74.7 (44.9)^a^	23.4 (18.1)^a^	55.4 (37.9)	42.8 (31.6)
*Note.* Standard deviations appear in parentheses.				
^a^ Denotes that conditions differ significantly at *p* < .001 (see main text).				

**Table 3 tbl3:** Study 2: Participant Characteristics by Condition

	Heightened ingratiation condition (*N* = 74)		Reduced ingratiation condition (*N* = 75)	
	Heavy drinking confederate (*N* = 36)	No drinking confederate (*N* = 38)	Heavy drinking confederate (*N* = 34)	No drinking confederate (*N* = 41)
Age (in years)	28.7 (11.9)	26. 4 (11.2)	25.5 (9.8)	25.4 (10.0)
Units per week	17.1 (9.9)	13.7 (7.5)	14.6 (9.5)	13.7 (8.8)
Gender	23 women, 13 men	25 women, 13 men	16 women, 18 men	28 women, 13 men
*Note*. Parentheses denote standard deviation for age and units per week*.*				

**Table 4 tbl4:** Study 2: Alcohol Consumption by Condition

	Heightened ingratiation condition (*N* = 74)		Reduced ingratiation condition (*N* = 75)	
	Heavy drinking confederate (*N* = 36)	No drinking confederate (*N* = 38)	Heavy drinking confederate (*N* = 34)	No drinking confederate (*N* = 41)
Grams alcohol consumed (*SD*)	7.8 (7.1)^a^	1.9 (4.6)^a^	5.7 (6.3)	3.0 (4.9)
*Note*. Standard deviations appear in parentheses*.*				
^a^ Denotes that conditions differ significantly at *p* < .001 (see main text).				

## Footnotes

1 Because we wanted to examine drinking behavior in a more naturalistic setting (mock bar) and a confederate ordering an alcohol drink but consuming very little of it may appear odd, we opted to instruct the confederate to only order a soft drink (hence the two confederate drinking conditions were heavy vs. no drinking).

2 An alternative analysis strategy is to discard the laboratory in which participants completed the study and conduct a 2 × 2 analysis of variance (ANOVA). The results and effect sizes do not significantly differ using this or the reported analysis approach.
