# Supplementary material for: Social Imitation of Alcohol Consumption and Ingratiation Motives in Young Adults
Source: Psychol Addict Behav. 2016 Jun;30(4):442–9. doi: 10.1037/adb0000150 (PMC4913807; doi:10.1037/adb0000150)
Supplement: Supplementary file 1 [file ze6002162984s1.doc]

**Supplemental Materials**

**Social Imitation of Alcohol Consumption and Ingratiation Motives in Young Adults**

**by E. Robinson et al., 2016, *Psychology of Addictive Behaviors***

**http://dx.doi.org/10.1037/adb0000150**

**STUDY 1: QUESTIONNAIRE RESPONSES**

Note: the term ‘other participant’ was used in the questionnaire measures which asked participants questions about the confederate’s behavior. When interpreting the responses to those questions we use the term ‘confederate’.

To probe participant suspicion concerning the behavior of the confederate and whether participants knew the confederate, participants rated on a 5-point Likert scale (strongly disagree to strongly agree) ‘I thought the other participant behaved oddly’ and ‘I have met the other participant before’. Mean responses on these items were 1.6 (SD = 0.7) and 1.3 (SD = 0.7), indicating that they did not believe the confederate had behaved oddly and did not know the confederate. Inclusion of these variables as covariates had no effect on any of the results reported.

Participants were asked whether they noticed how much the confederate drank (yes or no), whether the other confederate drank most of their drink and if the amount the confederate drank was odd (5-point Likert scales, strongly disagree to strongly agree). Participants disagreed when asked whether they thought the amount the confederate drank was odd (M = 1.98, SD = 0.9). 73/80 participants (91%) reported that they had noticed the amount the confederate drank. As expected the two conditions differed on their impressions of how much the confederate had consumed (p<.05), confirming that the manipulation was successful. In the light drinking condition participants disagreed when asked whether the confederate had consumed most of their drink (M = 1.4, SD = 0.5) and in the heavy drinking condition participants agreed (M = 4.7, SD = 0.6).

**STUDY 1: REMOVAL OF PARTICIPANTS**

With the 11 participants removed who came close to guessing the aims of the study **there was still a significant main effect of confederate drinking behavior [p = .001, *np^2^* = .16], no significant main effect of ingratiation motive [p = .90, *np^2^* < .01] and a significant interaction between confederate drinking behavior and ingratiation motives [p = .010, *np^2^* = .10]. As in the reported analyses, when participants drank under heightened ingratiation motives they drank significantly more (p < .001) in the presence of a heavy drinking confederate (M = 75.6, SD = 43.56) than in the presence of a light drinking confederate (M = 23.5, SD = 19.4). When participants drank under reduced ingratiation motives there was no significant difference between how much participants consumed in the heavy (M = 52.1, SD = 38.8) and light (M = 44.8, SD = 32.2) confederate drinking conditions (p = .99).**

**STUDY 2: QUESTIONNAIRE RESPONSES**

Embedded within the questions about their experience in the study (Questionnaire 2), participants completed a question (5-point Likert scale, strongly disagree to strongly agree) to measure whether the bogus participant grid manipulation was successful (‘I think the other participant liked me’). The two ingratiation conditions differed in how much they believed the confederate liked them (p < .001). Participants in the reduced ingratiation motive condition were significantly more likely to believe the confederate liked them (M = 3.91, SD = 0.5) than participants in the heightened ingratiation condition (M = 2.97, SD = 0.6).

In Questionnaire 3, participants were asked if they could remember the drinks the other participant had consumed (free text) and if they noticed the information recorded on the other participant’s (confederate) first questionnaire (free text). After this participants were provided with a blank version of Questionnaire 1 and asked to indicate which responses the other participant had made. Only two participants reported not being able to recall the drinks the confederate had chosen and with the exception of these two participants, participants were accurate in recalling whether the confederate had consumed alcoholic or non-alcoholic drinks. Of the 149 participants, 30 (20%) reported that they had not noticed the way the bogus questionnaire had been completed by the confederate or opted not to look at it. Of the participants who did report noticing the bogus questionnaire, when asked to reproduce the bogus questionnaire about the confederate’s initial views (on a 5-point Likert scale, strongly disagree to strongly agree), the two ingratiation motive conditions were accurate. Participants in the reduced ingratiation condition tended to indicate that the confederate had reported that they believed they could be friends (M = 4.5, SD = 0.6), would enjoy spending time together (M = 4.3, SD = 0.6) and that the confederate believed they (the participant) were interesting (M = 4.2, SD = 0.6). Conversely, participants in the heightened ingratiation condition tended to indicate the opposite. Participants reported that the confederate’s responses had indicated that they may not be friends (M = 2.3, SD = 0.6), may not enjoy spending time together (M = 2.5, SD = 0.6) and that the confederate was unsure how interesting the participant was (M = 2.9, SD = 0.6). As in Study 1, participants indicated that they did not know the confederate.

**STUDY 2: AGE**

Results of the 2x2x2 ANOVA for age indicated that there were no significant main effects of confederate drinking behavior **[F(1, 140) = 0.83, p = .37, *np^2^* = .006], ingratiation motive condition [F(1, 140) = 2.3, p = .14, *np^2^* = .016] and there were no significant interactions between confederate drinking behavior and ingratiation motive condition [F(1, 140) = 0.1, p = .74, *np^2^* = .001] or between confederate drinking behavior and laboratory type [F(1, 140) = 0.38, p = .54, *np^2^* = .003]. In addition, there was no significant three-way interaction [F(1, 140) = 0.39, p = .53, *np^2^* = .003]. There was however a significant main effect of laboratory type [F(1, 140) = 80.3, p <. 001, *np^2^* = .365]: participants in the bar laboratory (M age = 20.6, SD = 4.2) tended to be younger than participants in the normal laboratory (M age = 33.3, SD = 11.9). There was also a significant laboratory type and ingratiation motive condition interaction [F(1, 140) = 5.5, p= .021, *np^2^* = .038]. This interaction was explained by there being a larger age difference between the bar laboratory (M age = 20.0, SD = 2.4) vs. normal laboratory (M age = 36.1, SD = 11.8) in the heightened ingratiation motives condition, in comparison to the reduced ingratiation motives condition; bar laboratory (M age = 21.2, SD = 5.4) vs. normal laboratory (M age = 30.5, SD = 11.5).**

**To address this age imbalance, we examined whether age was associated with our dependent variable of interest (grams of alcohol consumed). There was no significant association between age and grams of alcohol consumed (r = -0.08, p = 0.35) in the total sample. Moreover, when included as a covariate in the 2x2x2 ANOVA, age was not significantly associated with alcohol consumption and none of the significant and non-significant results reported in the main analyses were affected. Thus, age appeared to have little or no effect on alcohol consumption and our main findings were not dependent on whether age was controlled for.**

**STUDY 2: ALCOHOL CONSUMPTION**

As noted in the manuscript, in the 2x2x2 ANOVA for alcohol consumption there was no significant main effect of laboratory type **[F(1, 141) = 2.1, p = .15, *np^2^* = .015], there was no significant interaction between laboratory type and ingratiation motive condition [F(1, 141) = 1.4, p = .24, *np^2^* = .010] and there was no significant three way interaction [F(1, 141) = 0.24, p = .63, *np^2^* = .002]. There was a significant laboratory type and confederate drinking behavior interaction [F(1, 141) = 9.3, p = .003, *np^2^* = .062]. This interaction was explained by participants in the heavy drinking confederate condition consuming significantly more alcohol (t (68) = 2.5, p = .016) in the bar lab (M = 8.5 grams, SD = 7.2) than in the normal lab (M = 4.6 grams, SD = 5.7), whereas the amount of alcohol consumed by participants in the no drinking confederate conditions did not differ across the bar lab (M = 1.8 grams, SD = 4.7) and normal lab (M = 3.3 grams, SD = 4.8); both conditions drank a similar amount of alcohol (t (77) = 1.4, p = .16).**

**Note: this effect is not directly related to our theoretical proposition. Importantly, there was no three-way interaction between laboratory type, ingratiation motives and confederate drinking behavior, suggesting that the effect that ingratiation motives has on social mimicry is not dependent on the laboratory type participants were tested in.**

**As the finding that participants drank more in the presence of a heavy drinking confederate in the bar lab vs. the normal lab was not hypothesised, we can only speculate that it may be the case that the behavior of the heavy drinking confederate (which may have indicated that it would be normal for participants to drink alcohol) and the setting (being in a bar lab may indicate that it is normal to drink alcohol) had an additive effect and made drinking alcohol appear particularly acceptable to the participants. However, because different confederates were used in the bar and normal laboratory sessions we cannot and do not make any firm conclusions concerning the cause or relevance of the laboratory type and confederate drinking behavior interaction.**

**STUDY 2: REMOVAL OF PARTICIPANTS**

In the analysis which did not include the 24 participants who came close to guessing the aims of the study, **there was still a significant main effect of confederate drinking behavior [p = .001, *np^2^* = .09], no significant main effect of ingratiation motive [p = .45, *np^2^* = .005] and the interaction between confederate drinking behavior and ingratiation motives approached significance [p = .089, *np^2^* = .025]. The interaction between laboratory type and confederate drinking behavior remained significant [p = .018, *np^2^* = .047] and the interaction between laboratory type and ingratiation motive remained non-significant [p = 0.21, *np^2^* = .014]. The three-way interaction between laboratory type, ingratiation motive and confederate drinking behavior also remained non-significant [p = .70, *np^2^* = .001].**

**Critically, with these 24 participants excluded, in the heightened ingratiation motive condition participants still drank significantly more with a heavy vs. no drinking confederate [p < .001], whilst in the reduced ingratiation motive condition there was still no significant effect of confederate behavior [p = .18]. See table below for means and standard deviations.**

|  | Heightened ingratiation condition  (*N*=63) | | Reduced ingratiation condition  (*N*=62) | |
| --- | --- | --- | --- | --- |
|  | Heavy drinking confederate (*N*=30) | No drinking confederate  (*N*=33) | Heavy drinking confederate  (*N*=28 ) | No drinking confederate  (*N*=34) |
| Grams alcohol  consumed (SD) | 7.6 (6.8) | 2.1 (4.9) | 5.7 (6.4) | 3.3 (4.9) |
